# The interaction between cancer associated fibroblasts and tumor associated macrophages via the osteopontin pathway in the tumor microenvironment of hepatocellular carcinoma

**DOI:** 10.18632/oncotarget.27881

**Published:** 2021-02-16

**Authors:** Kazunori Tokuda, Yuji Morine, Katsuki Miyazaki, Shinichiro Yamada, Yu Saito, Masaaki Nishi, Takuya Tokunaga, Tetsuya Ikemoto, Satoru Imura, Mitsuo Shimada

**Affiliations:** ^1^Department of Surgery, Institute of Biomedical Sciences, Tokushima University Graduate School, Tokushima 770-8503, Japan

**Keywords:** hepatocellular carcinoma, osteopontin, cancer associated fibroblast, tumor associated macrophage, tumor microenvironment

## Abstract

Background: Cancer-tumor associated macrophage (TAM)-cancer associated fibroblast (CAF) interactions are an important factor in the tumor microenvironment of hepatocellular carcinoma.

Materials and Methods: Hepatic stellate cells (HSCs) were cultured with cancer cell-conditioned medium (Ca.-CM), TAM-CM and CAF-CM, and the expression of CAF markers were evaluated by RT-PCR. Whether HSCs cultured with Ca.-CM, TAM-CM and CAF-CM contributed to the enhanced malignancy of cancer cells was examined using proliferation, invasion and migration assays. Furthermore, the differences between these three types of CM were evaluated using cytokine arrays.

Results: HSCs cultured with Ca.-CM, TAM-CM and CAF-CM showed significantly increased mRNA expression of αSMA, FAP and IL-6. All HSCs cultured with each CM exhibited significantly increased proliferation, invasion and migration of cancer cells. The osteopontin concentration was significantly higher in HSCs cultured with TAM-CM than the other CAF-CMs. Osteopontin inhibition significantly reduced osteopontin secretion from HSCs cultured with TAM-CM and suppressed the proliferation and invasion of cancer cells enhanced by HSCs cultured with TAM-CM.

Conclusions: We observed enhanced osteopontin secretion from TAMs, and this increased osteopontin further promoted osteopontin secretion from HSCs cultured with TAM-CM, leading to increased malignancy. For the first time, we demonstrated the importance of cancer-TAM-CAF interactions via osteopontin in hepatocellular carcinoma.

## INTRODUCTION

The tumor progression has been regarded as a multistep process involving genetic and epigenetic changes targeting only cancer cells. However, recent research has identified that the tumor microenvironment (TME) is an equally important factor of tumor behavior [1]. Tumor tissues mainly contain not only cancer cells but also fibroblasts, macrophages and vascular components that form the TME, which regulates interaction and differentiation induction.

Fibroblast is a multifunctional cell type in connective tissue that deposit basement membrane components and extracellular matrix (ECM) and regulate differentiation in associated epithelial cells. There are a large number of cancer associated fibroblasts (CAFs) in the TME [2, 3]. In addition to enhancing angiogenesis and the proliferation of cancer cells, CAFs have been implicated in enhancing cancer cells invasiveness, possibly through the induction of epithelial-mesenchymal transition (EMT) [4].

Cancer cells are affected by immune cells through all stages of the tumor, from the early stage to tumor progression and metastasis. In this regard, macrophages play a prominent role in and actively contribute to each stage of cancer [5]. Macrophages are attracted to and activated by TME-derived cytokines, which induce their differentiation into tumor associated macrophages (TAMs). It has been reported that TAMs support the increased malignancy of cancer cells and therapeutic resistance of multiple cancer types [2, 6–9]. TAMs can be classified into two types: 1) classically activated, proinflammatory “M1” and 2) alternatively activated, anti-inflammatory “M2”. Generally, during the early stages of tumor development, M1 macrophages are mainly responsible for the TH1 cell response by secreting pro-inflammatory cytokines. In the later stages of tumor progression and metastasis, M2 macrophages exhibit a low antigen presentation efficiency and produce high levels of anti-inflammatory cytokines [10–14]. In this study, M2 macrophages are termed TAMs.

We have previously reported the role of the transcriptional factor NF-E2-related factor 2 (Nrf2) in cancer-TAM interactions in hepatocellular carcinoma (HCC) and pancreatic cancer [15] and cancer-CAF interactions through IL-6 and CXCL10 in pancreatic cancer [16]. Considering our results and previous findings, CAFs and TAMs play a central role in the development of the TME, and the establishment of cancer-CAF-TAM interactions likely increase the malignancy of tumors. Li, et al. [17] reported that CAFs effectively attracted monocytes via the CXCL12/CXCR4 pathway and induced their differentiation into TAM in oral squamous cell carcinoma. Zhang, et al. [18] reported that IL-8 produced by CAFs attracted monocytes and promoted the polarization of M2 macrophages in colorectal cancer. However, there are limited reports on the relationship between CAFs and TAMs.

Therefore, this study particularly focused on the direct relationship between CAFs and TAMs in HCC. We showed that TAMs activated hepatic stellate cells, which promoted cancer proliferation and migration. Additionally, we identified osteopontin (OPN) as a key molecule involved in cancer-CAF-TAM interactions in HCC. OPN is an intracellular and secreted chemokine-like phosphorylated glycoprotein and is frequently increased in numerous human cancers. It is an important for the regulation of proliferation, invasion, metastasis, angiogenesis, stemness, inflammatory responses, ECM degradation. [19–23]. In this study, the function of OPN in the TME of HCC was elucidated.

## RESULTS

### HSCs were activated by the CM from cancer cells, TAMs and CAFs

**Figure 1:**
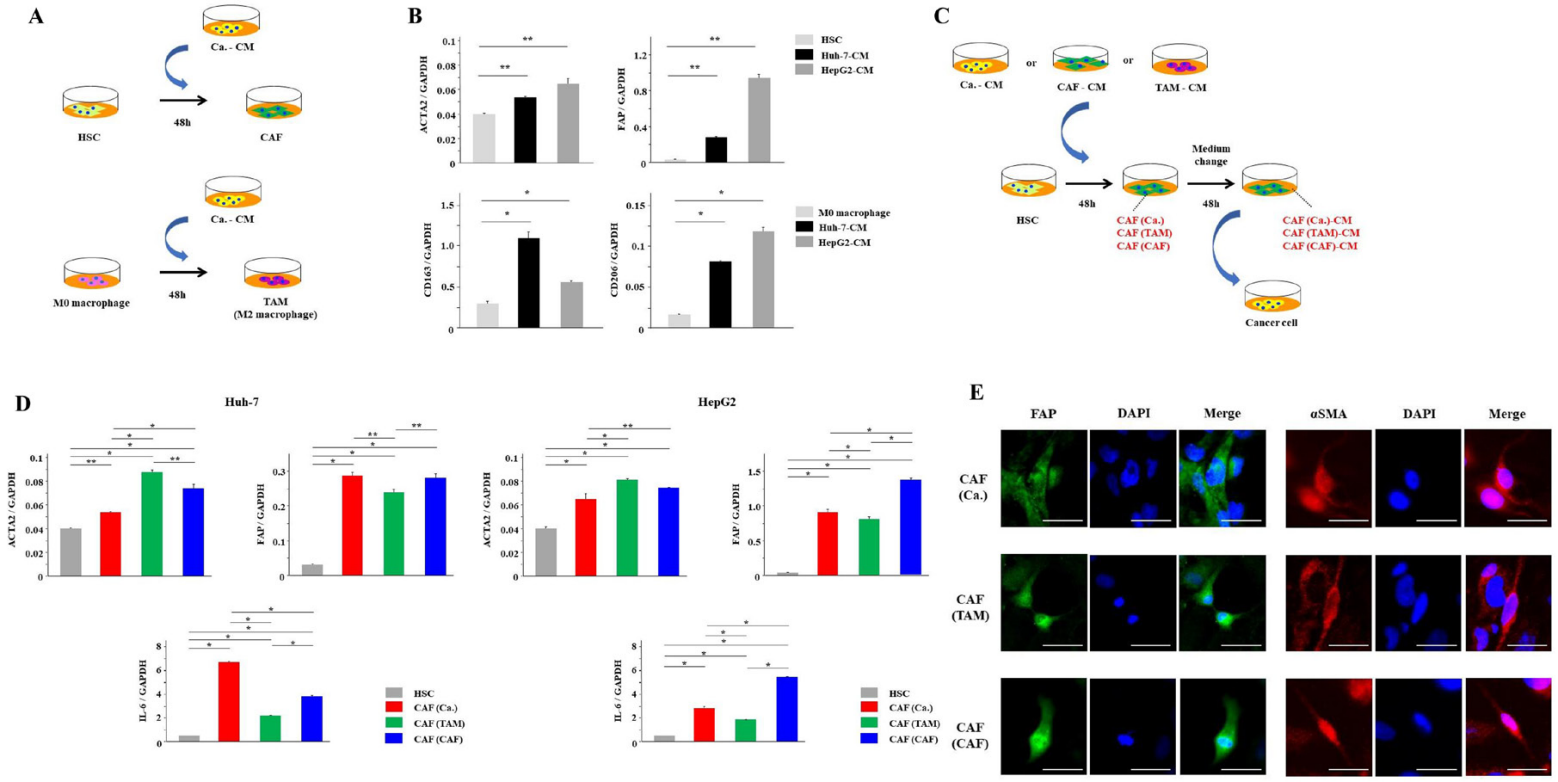
HSCs were activated by cancer cells, TAMs and CAFs. (**A**) The scheme of CAF and TAM induction from cancer cells. (**B**) HSCs and M0 macrophages were cultured with Ca.-CM for 48 hours. αSMA and FAP mRNA expression levels in HSCs, and CD163 and CD206 mRNA expression levels in macrophages cultured with Ca.-CM were analyzed by PCR analysis. (**C**) The scheme of CAF induction from cancer cells, TAMs and CAFs. (**D**) HSCs were cultured with Ca.-CM, TAM-CM and CAF-CM for 48 hours. αSMA, FAP and IL-6 mRNA expression in these HSCs were analyzed by PCR. (**E**) Immunofluorescence staining of FAP and αSMA in each CAF. Scale bar; 50 μm. The graphs in (B) and (D) show the data as the mean ± SD. ^*^ *P* < 0.01 and ^**^ *P* < 0.05 (one-way ANOVA with the Turkey-Kramer test, Mann-Whitney *U* test).

First, to induce the activation of HSCs into CAFs, we cultured HSCs with Ca.-CM for 48 hours (Figure 1A). Similarly, M0 macrophages were cultured with Ca.-CM for 48 hours to induce their differentiation into TAMs (Figure 1A). CAFs showed significantly increased mRNA expressions of CAF markers, such as αSMA and FAP (*P* < 0.05, Figure 1B), and TAMs exhibited significantly increased mRNA expressions of TAM markers, such as CD163 and CD206 (*P* < 0.05, Figure 1B). We then collected CAF-CM and TAM-CM. To investigate whether CAFs could also be induced by TAMs and CAFs, HSCs were cultured with TAM-CM or CAF-CM (Figure 1C). HSCs cultured with TAM-CM, CAF-CM or Ca.-CM showed significantly increased mRNA expression of αSMA, FAP and IL-6 (*P* < 0.05, Figure 1D). Immunofluorescence staining detected FAP and αSMA in each CAF. There was no difference in the morphology of each CAF (Figure 1E). Together, these results suggest that CAFs in the TME may originate from not only cancer cells but also TAMs and CAFs.

### All CAFs derived from cancer cells, TAMs, and CAFs enhanced the malignancy of cancer cells

**Figure 2:**
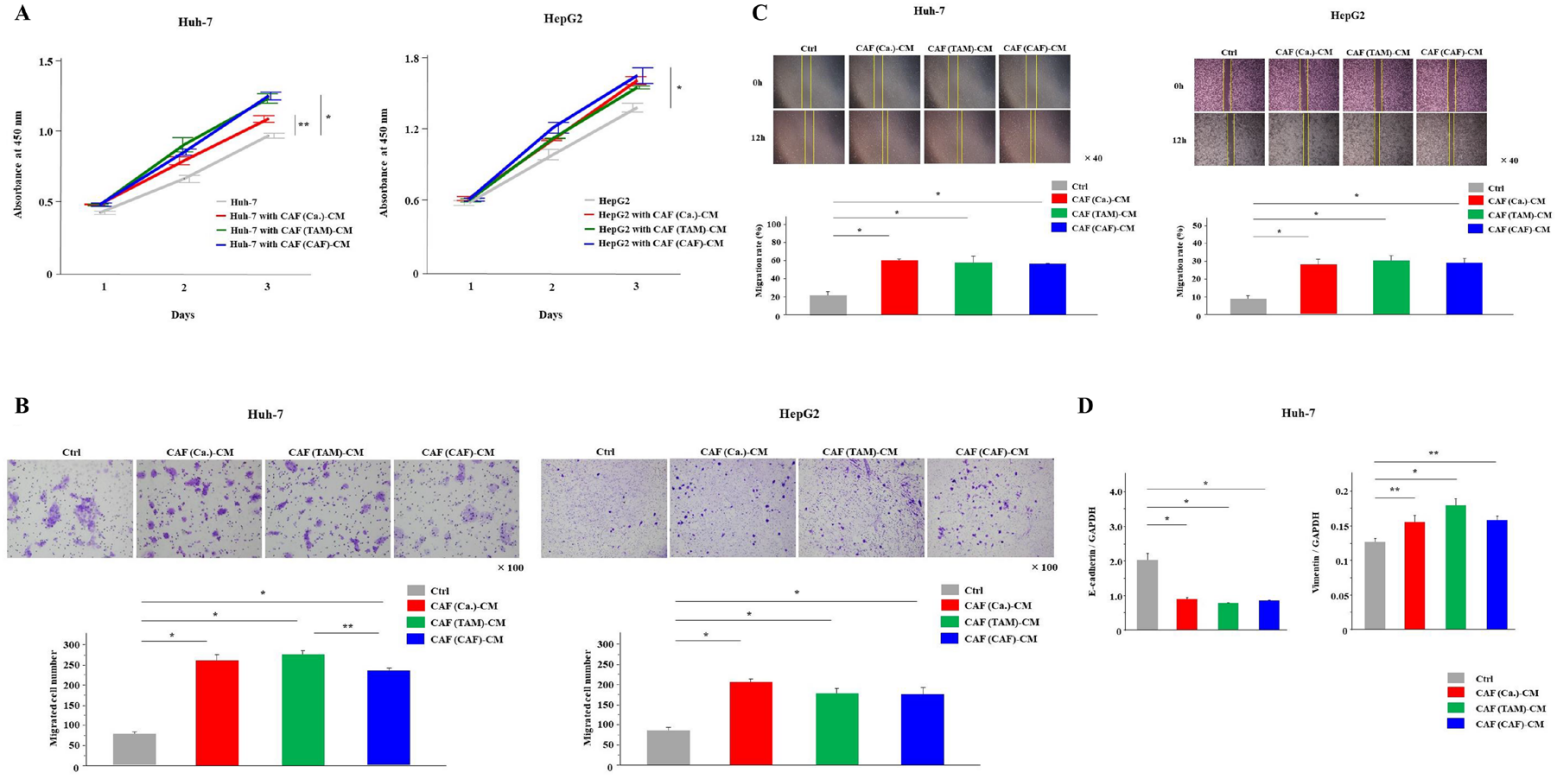
All CAFs derived from cancer cells, TAMs and CAFs enhanced cancer malignancy. (**A**–**C**) The (A) proliferation, (B) invasion and (C) migration of cancer cells with each CAF-CM or normal medium (control; Ctrl) were monitored for 3 days, 24 hours and 12 hours, respectively. (**D**) Cancer cells were cultured with each CAF-CM for 48 hours. E-cadherin and vimentin mRNA expression levels were analyzed by PCR analysis. The graphs show the data as the mean ± SD. ^*^ *P* < 0.01 and ^**^ *P* < 0.05 (one-way ANOVA with the Turkey-Kramer test).

We investigated the effects of CAFs on cancer cell malignancy. All three types of CAF-CMs were collected and added to cancer cells (Figure 1C). Compared with normal culture medium, all CAF-CMs increased the tumor grade, and significantly increased the proliferation, invasion and migration capabilities of cancer cells (*P* < 0.05, Figure 2A–2C). In cancer cells cultured with each CAF-CM, the mRNA expression of vimentin, which was the EMT marker, was significantly increased, and the mRNA expression of E-cadherin was significantly decreased (*P* < 0.05, Figure 2D).

### Only CAFs derived from TAMs secreted an increased level of OPN

**Figure 3:**
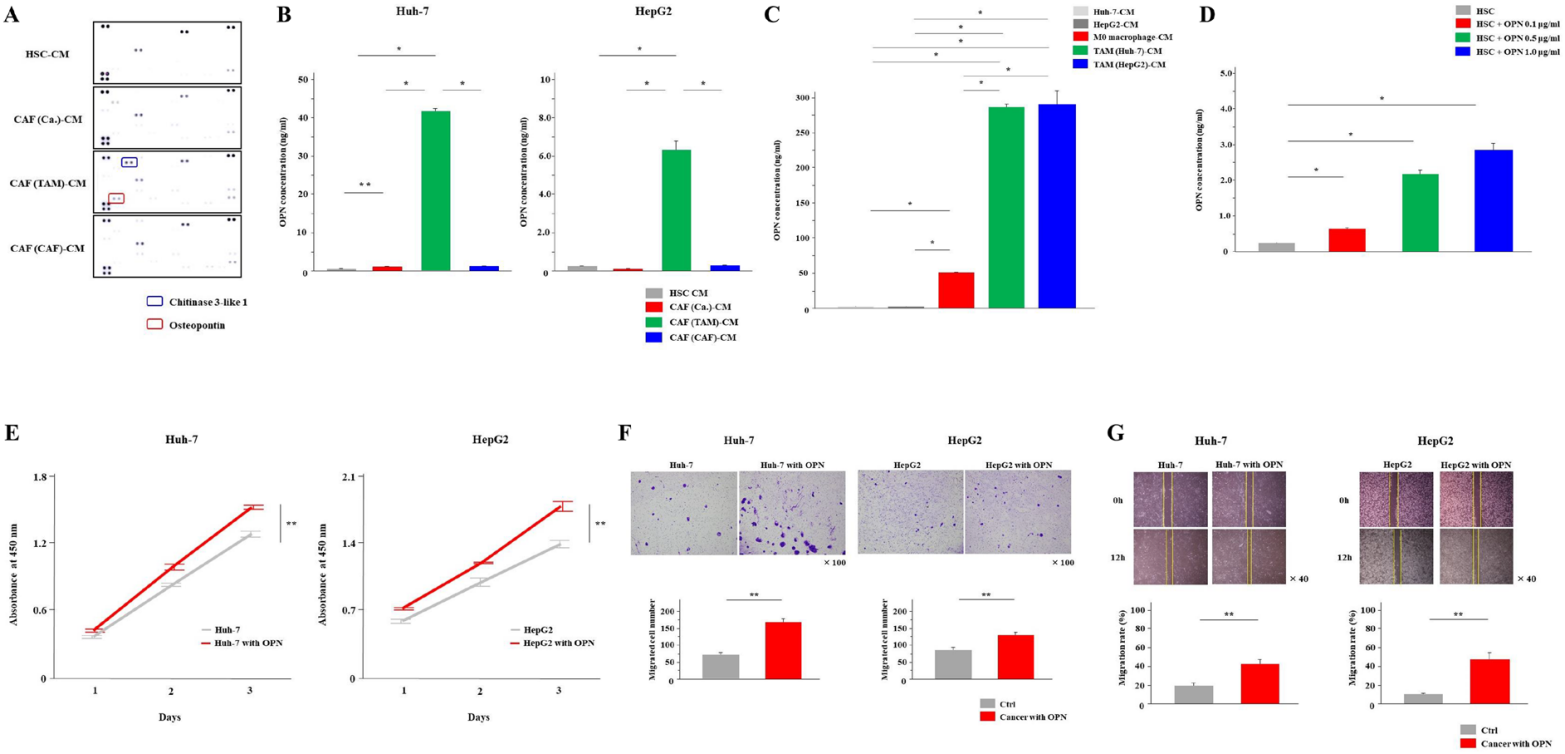
CAFs (TAM) secreted a significantly higher amount of osteopontin than other CAFs. (**A**) Cytokine arrays of CAF (Ca.), CAF (TAM) and CAF (CAF) culture supernatants. HSC-CM was used as the control. (**B**) OPN secretion from all CAFs was analyzed by ELISA. (**C**) The secretion of OPN from cancer cells, M0 macrophages and TAMs was examined by ELISA. (**D**) After adding OPN to HSCs at concentrations of 0.1, 0.5 and 1.0 μg/mL, the medium was exchanged once and the CM was collected. The secretion of OPN from HSCs was examined by ELISA. (**E**–**G**) The (E) proliferation, (F) invasion and (G) migration of cancer cells with the addition of the OPN at a concentration of 1.0 μg/mL or normal medium (control; Ctrl) were monitored for 3 days, 24 hours and 12 hours, respectively. The graphs show the data as the mean ± SD. ^*^ *P* < 0.01 and ^**^ *P* < 0.05 (one-way ANOVA with the Turkey-Kramer test, Mann-Whitney *U* test).

Next, we performed cytokine arrays of these three types of CAF culture supernatants to examine the differences in cytokine secretion. The results showed that the secretion of OPN and chitinase 3-like 1 was increased in CAF (TAM)-CM (Figure 3A). OPN is an integrin-binding glycophosphoprotein and has been reported to be associated with cancer malignancy. Therefore, we focused on OPN and conducted the following study. The ELISA results also revealed that the OPN concentration was significantly higher in CAF (TAM)-CM than the other CAF-CMs (*P* < 0.01, Figure 3B). In addition, when the secretion of OPN from cancer cells, M0 macrophages and TAMs was examined by ELISA, significantly increased OPN secretion from TAMs was observed (*P* < 0.01, Figure 3C). When HSCs were cultured with OPN added, the secretion of OPN from HSCs was enhanced in a concentration-dependent manner (*P* < 0.01, Figure 3D). From these findings, only CAFs (TAM) had the ability to secret OPN, which may depend on the OPN secreted from TAMs. Next, the effect of OPN on cancer cells was examined. The addition of the OPN at a concentration of 1.0 μg/mL significantly enhanced the proliferation, invasion and migration capabilities of cancer cells (*P* < 0.05, Figure 3E–3G).

### Inhibition of OPN suppressed the proliferation, invasion and migration of cancer cells induced by CAFs derived from TAMs

**Figure 4:**
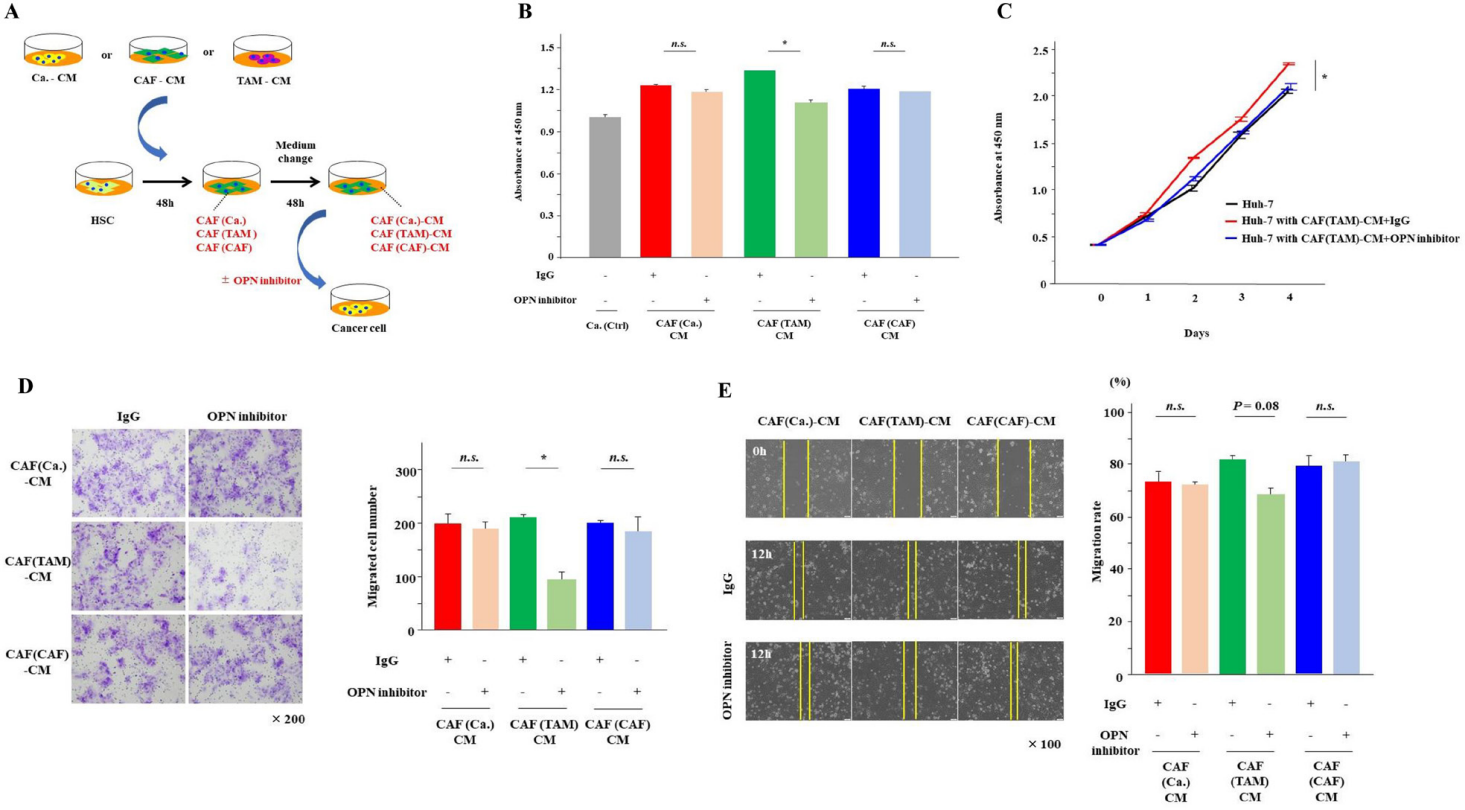
Inhibition of OPN suppressed cancer proliferation, invasion and migration. (**A**) The scheme of the experiment examining the effects of OPN inhibitors on cancer malignancy. Cancer cells were cultured for 48 hours with each CAF-CM in the presence or absence of the OPN antibody. (**B**) The proliferation of cancer cells cultured in each CAF-CM with or without the OPN antibody was analyzed at day 2. (**C**) The proliferation of cancer cells cultured in CAF(TAM)-CM with or without the OPN antibody was monitored for 4 days. (**D**) The invasion of cancer cells cultured in each CAF-CM with or without the OPN antibody for 24 hours was analyzed. (**E**) The migration of cancer cells cultured in each CAF-CM with or without the OPN antibody for 12 hours was analyzed. The graphs show the data as the mean ± SD. ^*^ *P* < 0.01 and ^**^ *P* < 0.05 (one-way ANOVA with the Turkey-Kramer test).

To investigate the effects of OPN on cancer cells, the examination from here was carried out using Huh-7 cells. Huh-7 cells were cultured for 48 hours in the three types of CAF-CMs with and without the OPN antibody (Figure 4A). The addition of the OPN antibody significantly suppressed the proliferation and invasion of Huh-7 cells that were enhanced by CAF (TAM)-CM (*P* < 0.01). Although there was no significant difference, OPN neutralization tended to suppress the migration of Huh-7 cells enhanced by CAF (TAM)-CM (*P* = 0.08). These effects of the OPN antibody were observed only when cultured with CAF (TAM)-CM, and no inhibitory effects were observed when Huh-7 cells were cultured with the other CAF-CMs (Figure 4B–4E).

### OPN inhibition reduced the OPN secretion from CAFs derived from TAMs

**Figure 5:**
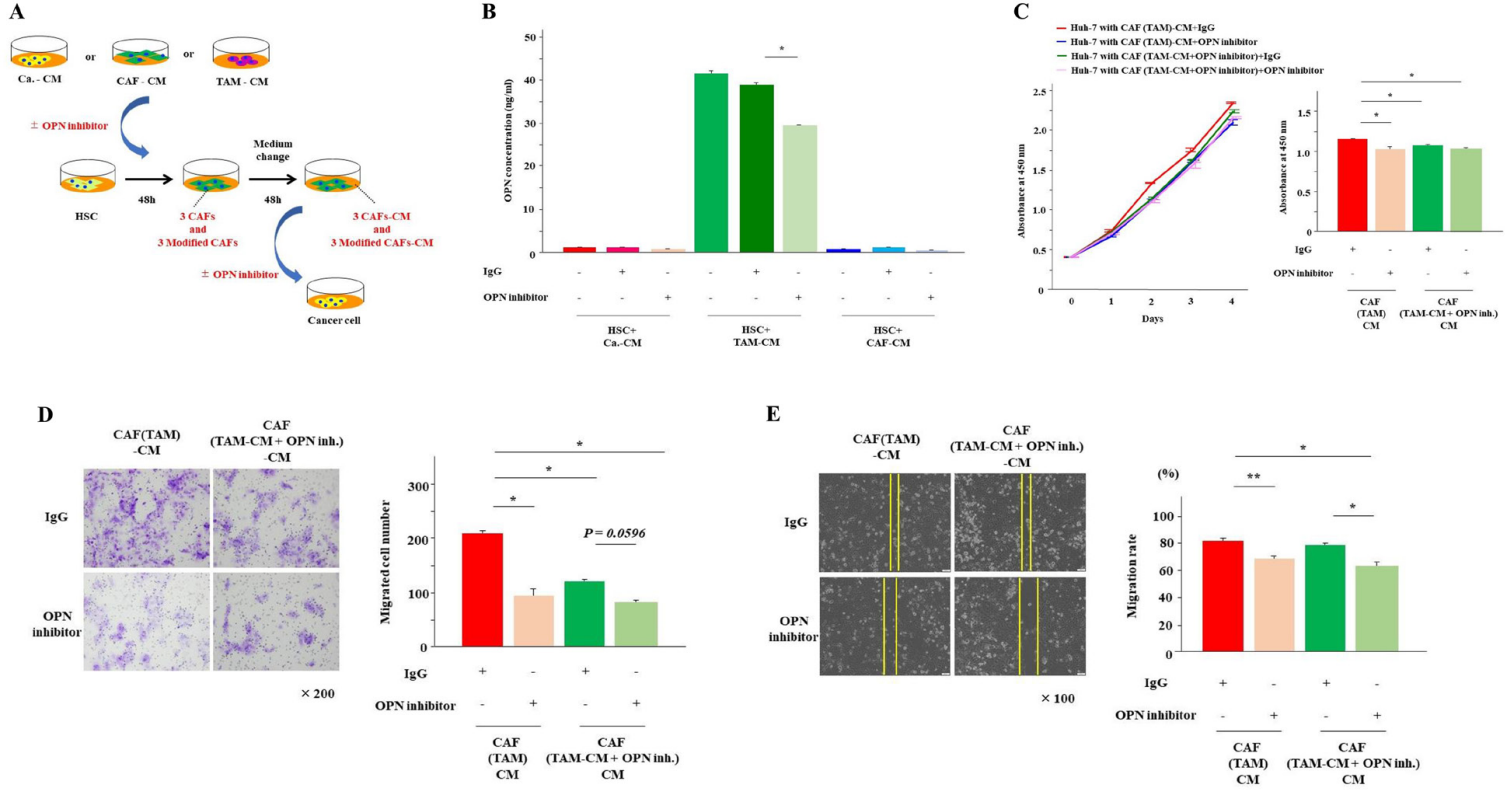
OPN inhibition reduced OPN secretion from CAFs (TAM). (**A**) The scheme of the experiment examining the effects of OPN inhibitors on CAFs. HSCs were cultured for 48 hours with each CAF-CM (Ca.-CM, TAM-CM and CAF-CM) in the presence or absence of the OPN antibody. (**B**) The secretion of OPN from CAFs cultured in each CM with or without the OPN antibody for 48 hours was examined by ELISA. (**C**) The proliferation of cancer cells cultured in CAF (TAM)-CM with or without the OPN antibody and CAF (TAM-CM+OPN inhibitor)-CM, which was prepared by culturing HSCs with TAM-CM in the presence or absence of the OPN antibody for 48 hours, was monitored for 4 days and analyzed at day 2. (**D**) The invasion of cancer cells in CAF (TAM)-CM with or without the OPN antibody and CAF (TAM-CM+OPN inhibitor)-CM with or without the OPN antibody for 24 hours was analyzed. (**E**) The migration of cancer cells in CAF (TAM)-CM with or without the OPN antibody and CAF (TAM-CM+OPN inhibitor)-CM with or without the OPN antibody for 12 hours was analyzed. The graphs show the data as the mean ± SD. ^*^ *P* < 0.01 and ^**^ *P* < 0.05 (one-way ANOVA with the Turkey-Kramer test).

Next, to examine the effect of OPN on CAFs, HSCs were cultured for 48 hours in the presence of Ca.-CM, CAF-CM and TAM-CM with and without the OPN antibody when inducing the three types of CAFs (Figure 5A). As a result, almost no OPN was detected in CAFs cultured in Ca.-CM and CAF-CM, regardless of the presence or absence of the OPN antibody. However, the secretion of OPN was significantly reduced in CAFs cultured with TAM-CM (Figure 5B). When Huh-7 cells were cultured with CAF (TAM)-CM in the presence of the OPN antibody, their proliferation and invasion abilities were significantly reduced (Figure 5C and 5D). In contrast, there was no significant difference in migration (Figure 5E).

### OPN positive CAFs were expressed in the tissues of HCC patients

**Figure 6:**
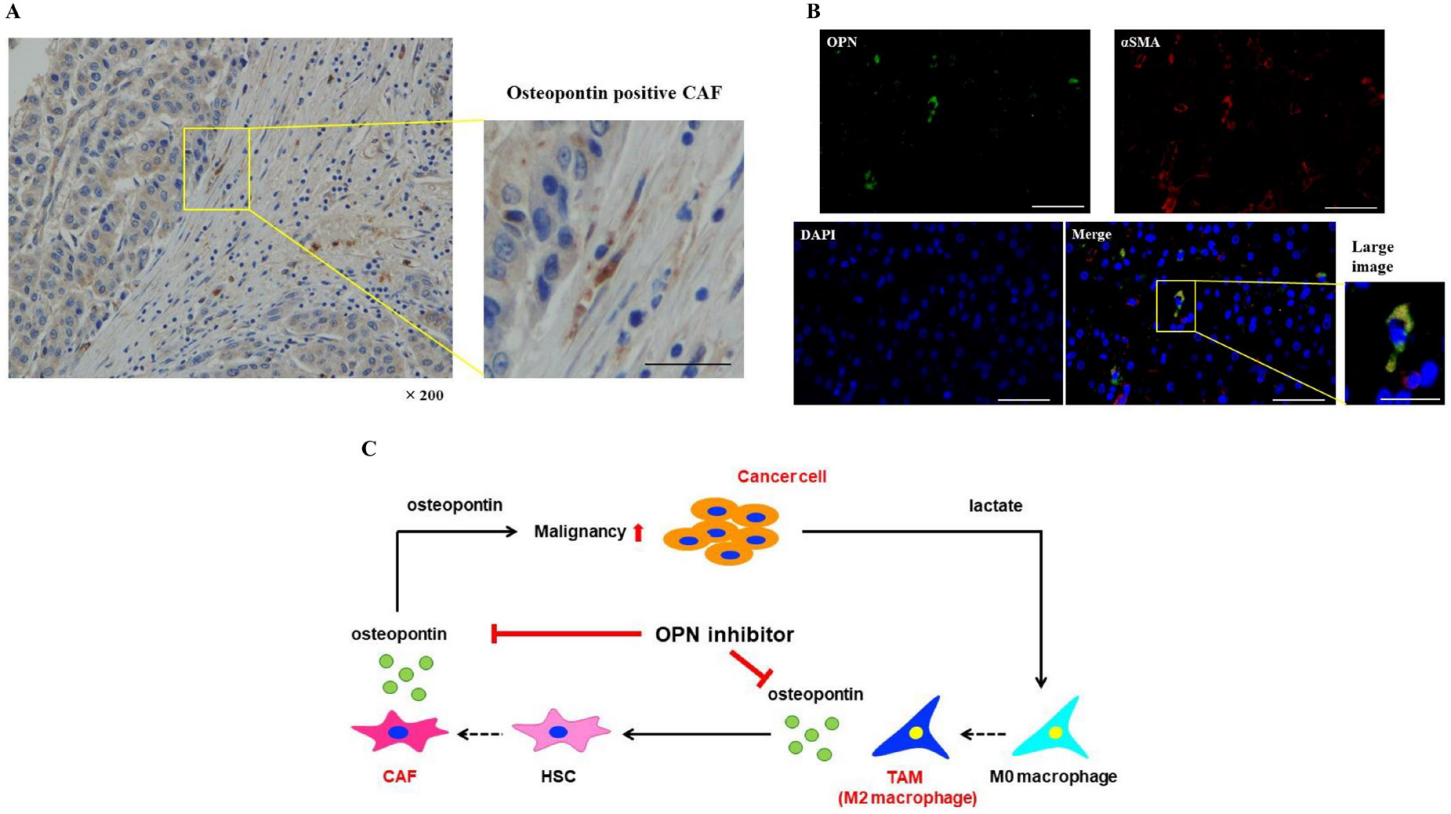
OPN positive CAFs were expressed in the tissues of HCC patients. (**A**) Immunohistochemical staining of OPN in the tissues of HCC patients. Scale bar; 50 μm. (**B**) Double immunofluorescence was carried out in HCC tissues. OPN expression in the cells was indicated in green, αSMA positivity was indicated in red, and OPN plus αSMA positivity was indicated in yellow. Scale bar; 50 μm, scale bar of large image; 25 μm. (**C**) The proposed model of cancer-TAM-CAF interactions via OPN.

Immunohistochemical staining revealed OPN positive spindle shaped cells in the tissues of HCC patients (Figure 6A). In addition, double immunofluorescence was carried out in HCC tissues. OPN expression in the cells was indicated in green, αSMA positivity was indicated in red, and OPN plus αSMA positivity was indicated in yellow. OPN and αSMA strongly positive CAFs were observed in HCC tissues (Figure 6B).

## DISCUSSION

In this study, we hypothesized that CAFs might be derived from not only cancer cells but also TAMs or CAFs in the TME, and that there may be differences among these three types of CAFs. We found two novel findings regarding CAF characteristics. First, CAFs could be derived from the CM of cancer cells, TAMs and CAFs. Second, OPN was identified as a key molecule secreted from only CAFs induced by TAM-CM, and this OPN played an important role in enhancing the malignancy of cancer cells via cancer-CAF-TAM interactions.

Teng, et al. [24] reported that CAFs promoted cancer progression via the SDF-1/CXCR4 axis in endometrial cancer, and Jia, et al. [25] reported that hepatocyte growth factor secreted by CAFs played a key role in HCC proliferation. There have been many reports that cancer-educated fibroblasts contribute to the increased malignancy of tumors [24–34]. Cancer cells and CAFs release similar inflammatory cytokines such as IL-6 and TNF-α, and it is considered reasonable that CAFs are induced by Ca.-CM and CAF-CM. However, there are few reports that macrophages activate fibroblasts. Ueshima, et al. [35] demonstrated that TGF-β1 secreted from macrophages promoted fibroblast differentiation and scar formation in the ureter. To the best of our knowledge, this study is the first to confirm the activation of CAFs by TAMs in the TME. In the present results, CAFs (TAM) showed the highest mRNA expression of αSMA, but mRNA expression of IL-6 was significantly lower than that of other CAFs. This is because the phenotype of CAF (TAM) is CAF-like, but its functionality may be different from other CAFs like the difference in OPN. More detailed investigation is needed to clarify this difference.

Cho, et al. [11] reported that CAFs played a key role in polarization of TAM via the increased secretion of IL6 and GM-CSF in response to cancer cell stimulation. Andersson, et al. [36] reported that CAFs secreted high levels of IL-33, which induced TAMs to undergo M1 to M2 transition, and they provided mechanistic insight into the IL-33/NF-κB/MMP9/laminin axis that mediates CAF-TAM-committed cancer metastasis. However, the detailed mechanisms underlying cancer-CAF-TAM interactions have not been fully elucidated.

To investigate the difference in cytokine release from the three types of CAFs, which were derived from cancer cells, TAMs and CAFs, we performed a cytokine array for each CM. As a result, OPN secretion was identified as the characteristic difference between the three types of CAFs in this study. OPN was first reported as a phosphoprotein secreted by transformed, malignant epithelial cells [37]. OPN binds to integrin and CD44 receptors to mediate cellular signaling and cell-matrix interactions [38]. OPN has been identified as a key non-collagenous bone matrix protein, and it plays a prominent role in diverse systems, such as immune and vascular systems. OPN regulates the production of cytokine and cell trafficking and inhibits ectopic mineralization and macrophage accumulation in vascular and immune systems, respectively [39]. It also mediates cell migration, adhesion [40] and, the production of cytokine in macrophages and acts as a survival factor [41]. Furthermore, OPN has been reported to contribute to the promotion of various cancers, such as HCC [42–45] and intrahepatic cholangiocarcinoma [23].

Regarding the mechanism of increased OPN secretion, Qin, et al. [46] reported that IL-6 secreted by CAFs was the main upstream molecule triggering the induction of neoplastic OPN in head and neck cancer. Konno, et al. [47] reported that the expression of OPN in monocytes and monocyte-derived dendric cells was induced by IL-10. In addition, Zhang, et al. [48] reported that the mRNA expression of OPN and OPN protein level in TAM were enhanced compared to M0 macrophage in lung cancer, supporting our result of increased OPN secretion from TAM. However, there are few reports on the mechanism contributing to the enhanced secretion of OPN from fibroblasts. Shimodaira, et al. [49] reported that the secretion of OPN from macrophages upregulated OPN expression in fibroblasts, which supports our results. Therefore, in the TME, OPN secreted from TAMs might enhance the secretion of OPN from CAFs. Furthermore, we confirmed that the inhibition of OPN suppressed the malignancy of cancer cells enhanced by culture with CAF (TAM)-CM. It was suggested that upregulated OPN contributed to the enhancement of cancer malignancy in HCC. Specifically, lactate produced by cancer cells induced M0 macrophages to polarize into TAMs as we previously reported [15], which increased the mRNA and protein expression of OPN and promoted the OPN secretion. The OPN secreted from TAMs enhanced the OPN secretion from CAFs, which subsequently increased the malignancy of cancer cells. It is also possible that OPN, which was highly secreted from TAMs, enhanced the malignancy of cancer. For the first time, we report that OPN is a key molecule for cancer-CAF-TAM interactions in the TME of HCC.

In this study, our results demonstrated that OPN was involved in the enhancement of cancer proliferation, invasion and migration. However, as a limitation to this study, the mechanisms underlying the increased secretion of OPN from TAMs and the OPN-induced malignancy of HCC cells were not elucidated. In addition, the findings were not investigated *in vivo*. Further considerations are required in the future.

In conclusion, we identified that the release of OPN from TAMs in the TME was increased, and this OPN further promoted the secretion of OPN from CAFs (TAM), leading to increased cancer cell malignancy (Figure 6C). In this study, we demonstrated the importance of cancer-TAM-CAF interactions via OPN for the first time. It has been suggested that OPN inhibitors might be useful in the prevention and treatment of hepatic inflammation and fibrosis in NASH [50]. Therefore, OPN might have potential as a new therapeutic target to inhibit cancer-CAF-TAM interactions in HCC.

## MATERIALS AND METHODS

### Cell culture

The HCC cell lines, Huh-7 and HepG2 were bought from the Riken Cell Bank (Tsukuba, Japan). Cancer cells were cultured in DMEM (Life Technologies Japan Ltd., Tokyo, Japan) containing 10% FBS (Life Technologies Japan Ltd., Tokyo, Japan). The human monocyte cell line THP-1 was bought from the Culture Collections of Public Health England and grown in RPMI-1640 (Wako, Osaka, Japan) containing 10% FBS. THP-1 cells were treated with 150 nM phorbol-12-myristate-13-acetate (PMA) (Sigma, St. Louis, MO, USA) for 48 hours to induce macrophages. The LX2 human hepatic stellate cell (HSC) line was obtained from Sigma-Aldrich. HSCs were cultured in DMEM containing 10% FBS. These cell lines were passaged for fewer than 6 months after resuscitation.

### Preparation of conditioned medium (CM)

Cancer cells were cultured in a 10-cm dish to 80% confluency. The cells were washed with PBS and then incubated with FBS-free medium. After 48 hours of incubation, the medium was collected, centrifuged (2000 rpm, 10 minutes) and filtered using a 0.2-μm filter to obtain cancer cell conditioned medium (Ca.-CM). The CM was used without additional FBS. CAF-derived cancer [CAF (Ca.)] and TAM-derived cancer media were prepared by adding Ca.-CM to HSCs or M0 macrophages for 48 hours (Figure 1A). Then, the medium was exchanged once, and the supernatant was collected. These supernatants were used as CAF conditioned medium (CAF-CM) and TAM conditioned medium (TAM-CM). CAFs derived from TAM-CM [CAFs (TAM)] and CAF-CM [CAFs (CAF)] were prepared in the same way (Figure 1C). To neutralize OPN in the CM, an OPN antibody (AF1433; R&D Systems, Inc., MN, USA) was added at a concentration of 1.0 μg/mL. In the examination of adding OPN, OPN (1433-OP; R&D Systems, Inc., MN, USA) was added to HSCs at concentrations of 0.1, 0.5 and 1.0 μg/mL, the medium was exchanged once in the same manner as above, and the CM was collected.

### Cell proliferation assay

Cell proliferation was investigated using a cell counting kit-8 (CCK-8) (Dojindo Molecular Technologies, Inc., Tokyo, Japan) in accordance with the manufacturer’s protocol. Briefly, cells were incubated with 10% CCK8-CM for 2 hours. Sample plates were used to measure the optical absorbance at 450 nm. The optical absorbance was measured using a SpectraMax i3 (Molecular Devices, LLC, San Jose, CA, USA) and SoftMax Pro 7 (Molecular Devices, LLC.).

### Migration assay

Transwell inserts (Corning, NY, USA) with an 8-μm pore size were used for migration assays. Cancer cells (2.0 × 10^4^) were seeded in the upper chamber. After cell attachment, the medium in the upper chambers was removed and fresh medium containing 1% FBS was added. Each CM containing 10% FBS was added to the lower chamber. After 24 hours incubation, the cells on the bottom of transwell inserts were fixed in 4% paraformaldehyde and stained with 0.2% crystal violet. The stained cells in three random microscopic fields (×100) were counted.

### Scratch assays

For scratch assays, cancer cells were seeded at a density of 2.0 × 10^4^ cells/well in 6-well plates. Once the cells were grown to confluency, a plastic pipette tip was scraped across the center of the well to produce a 1-mm wide wound area. The medium was removed, and each CAF-CM with or without the OPN antibody (AF1433) was added, and cancer cells were cultured for 12 hours. After culturing, a phase-contrast microscope was used to examine cell movement into the wound area.

### Cytokine array

The supernatants of LX2 cells, CAFs (ca.), CAFs (TAM) and CAFs (CAF) were collected, and the particulates were removed by filtration through a 0.2 μm filter. Cytokines in the supernatants were detected with a Proteome Profiler Human Cytokine Array Kit (ARY005B; R&D Systems, Inc., MN, USA), and membranes were developed following the manufacturer’s protocol. After blocking, membranes were incubated with the samples and antibody cocktail overnight at 4°C. After incubation, the membranes were washed and then incubated with streptavidin-HRP at room temperature for 30 minutes. Chemiluminescent detection reagents were incubated with the membrane for 1 minute, and the signal intensities on the membranes were detected with chemiluminescence (GE Healthcare, Little Chalfont, UK).

### Enzyme-linked immunosorbent assay

The level of OPN was detected using a human osteopontin Quantikine ELISA kit (DOST00; R&D Systems) in accordance with the manufacturer’s protocol. Absorbance at 450 nm was measured using a plate reader (SpectraMax i3; Molecular Devices) with a reference wavelength at 540 nm.

### Polymerase chain reaction analysis

The total ribonucleic acid (RNA) in each sample was extracted using a RNeasy Mini Kit (Qiagen, Hilden, Germany) in accordance with the manufacturer’s instructions. cDNA was synthesized using a reverse transcription kit (Applied Biosystems, Thermo Fisher Scientific Inc., Waltham, MA, USA). The following primers from TaqMan gene expression assays (assay identification number) were used: ACTA2 (Hs00426835_m1), FAP (Hs00990791_m1), IL6 (Hs00985639_m1), CD163 (Hs00174705_m1), CD206 (Hs00267207_m1), CDH1 (Hs01023894_m1) and VIM (Hs00185584_m1). GAPDH (4326317E) was selected as an internal control. The StepOnePlus Real-Time PCR System (Applied Biosystems) was used to perform RT-qPCR.

### Immunohistochemistry

We used immunohistochemistry procedures in our department that were previously reported [51]. Anti-OPN antibody (dilution 1:100, ab8448; Abcam plc., Cambridge, UK) was used as the primary antibody. This study was approved by Tokushima University Hospital ethics committee and with the approval of corresponding regulatory agencies, and all the experiments were carried out in accordance with the approved guidelines (Tokushima Clinical Trial Management System Number; 3215). All the patients involved in this study signed informed consent forms and agreed to participate.

### Immunofluorescence

We used immunofluorescence procedures in our department that were previously reported [52]. Anti-OPN antibody (dilution 1:1000, ab8448; Abcam plc.), Anti-αSMA antibody (dilution 1:100, ab7817; abcam plc.), and Anti-FAP antibody (dilution 1:1000, ab28244; abcam plc.) was used as the primary antibody. Anti-rabbit Alexa 488 (1:500, A-11008; Thermo Fisher Scientific, Inc.) for Anti-OPN antibody and Anti-FAP antibody, and Anti-mouse Alexa 594 (1:500, A-11005; Thermo Fisher Scientific, Inc.) for Anti-αSMA antibody were used as the secondary antibody.

### Statistical analysis

All statistical analyses were performed using statistical software (JMP software, version 13; SAS Campus Drive, Cary, NC). All data were expressed as the mean ± SD. Comparisons between 2 groups were performed by the Mann-Whitney *U* test. Comparisons between more than 3 groups were calculated using one-way ANOVA with the Turkey-Kramer’s test. A value of *P* < 0.05 was considered to indicate statistical significance.

## Abbreviations

α-SMA: α-smooth muscle actin
CAF: cancer-associated fibroblast
CCK8: cell-counting kit-8
CM: conditioned medium
DMEM: Dulbecco's Modified Eagle Medium
ECM: extracellular matrix
EMT: epithelial-mesenchymal transition
FAP: fibroblast activation protein
FBS: fetal bovine serum
GAPDH: gyceraldehyde-3-Phosphate Dehydrogenase
HCC: hepatocellular carcinoma
HSCs: hepatic stellate cells
IgG: immunoglobulin G
IL-6: interleukin-6
mRNA: messenger RNA
Nrf2: nuclear factor erythroid 2-related factor 2
OPN: osteopontin
TAM: tumor-associated macrophage
